# The Role of Glucocorticoids in the Treatment of ARDS: A Multicenter Retrospective Study Based on the eICU Collaborative Research Database

**DOI:** 10.3389/fmed.2021.678260

**Published:** 2021-07-26

**Authors:** Luming Zhang, Zichen Wang, Fengshuo Xu, Yinlong Ren, Hao Wang, Didi Han, Jun Lyu, Haiyan Yin

**Affiliations:** ^1^Intensive Care Unit, The First Affiliated Hospital of Jinan University, Guangzhou, China; ^2^Department of Clinical Research, The First Affiliated Hospital of Jinan University, Guangzhou, China; ^3^Department of Public Health, University of California, Irvine, Irvine, CA, United States; ^4^School of Public Health, Xi'an Jiaotong University Health Science Center, Xi'an, China; ^5^Department of Statistics, Iowa State University, Ames, IA, United States

**Keywords:** ARDS, glucocorticoids, ICU mortality, joint model, marginal structural cox model

## Abstract

**Background:** Acute respiratory distress syndrome (ARDS) is a common cause of respiratory failure in patients in intensive care unit (ICU). The therapeutic value of glucocorticoids (GCs) in the prognosis of ARDS remains controversial. The aim of this research is studying the impacts of GCs treatment on ARDS patients in ICU.

**Methods:** We retrospectively studied 2,167 ARDS patients whose data were collected from the public eICU Collaborative Research Database, among which 254 patients who received glucocorticoid (GCs) treatment were 1:1 matched by propensity matching analysis (PSM). The primary outcome was ICU mortality. Every oxygenation index (PaO2/FiO2) measurement before death or ICU discharge was recorded. A joint model (JM) which combined longitudinal sub-model (mixed-effect model) and time-to-event sub-model (Cox regression model) by trajectory functions of PaO2/FiO2 was conducted to determine the effects of GCs treatment on both ICU mortality and PaO2/FiO2 level and further PaO2/FiO2's effect on event status. The marginal structural cox model (MSCM) adjusted the overall PaO2/FiO2 of patients to further validate the results.

**Results:** The result of the survival sub-model showed that GCs treatment was significantly associated with reduced ICU mortality in ARDS patients [HR (95% CI) = 0.642 (0.453, 0.912)], demonstrating that GCs treatment was a protective factor of ICU mortality. In the longitudinal sub-model, GCs treatment was not correlated to the PaO2/FiO2. After adjusted by the JM, the HR of GCs treatment was 0.602 while GCs was still not significantly related to PaO2/FiO2 level. The JM-induced association showed that higher PaO2/FiO2 was a significant protective factor of mortality in ARDS patients and the HR was 0.991 which demonstrated that one level increase of PaO2/FiO2 level decreased 0.9% risk of ICU mortality. MSCM results also show that GCs can improve the prognosis of patients.

**Conclusion:** Rational use of GCs can reduce the ICU mortality of ARDS patients in ICU. In addition to the use of GCs treatment, clinicians should also focus on the shifting trend of PaO2/FiO2 level to provide better conditions for patients' survival.

## Introduction

Acute respiratory distress syndrome (ARDS) has a high incidence and is one of the most common severe diseases in intensive care unit (ICU) (1, 2), which is a manifestation of lung parenchymal disease and represents various serious conditions, ranging from transient dyspnea to rapid respiratory failure (3). In the United States, about 200,000 patients are diagnosed with ARDS each year, and about 75,000 of them die. Globally, ARDS affects 3 million people every year, accounting for 10% of the ICU and 24% of the mechanically ventilated patients in the ICU (4). A vital early step in the inflammatory response to ARDS is recruiting macrophages (5), which then combine with the vascular endothelium to penetrate the vascular wall (pulmonary microvascular endothelial cells) and tissue (6), leading to extravascular accumulation of protein-filled edema fluid, which is a key pathophysiological mechanism of ARDS. Glucocorticoids (GCs) counteract lung injury by improving epithelial permeability, reducing edema, inhibiting local, systemic inflammation and reducing apoptosis (7). However, GCs use may also cause immunosuppression and drug resistance (8). Whether corticosteroids improve the prognosis of ARDS remains controversial (9), especially regarding their dosage and duration. At present, a large number of studies are further verifying the relationship between GCs and ARDS (10, 11). A randomized trial by Tomazini et al. showed that the use of intravenous dexamethasone can increase the survival days of patients with COVID-19 and moderate or severe ARDS (12). Another randomized study showed that dexamethasone reduced 28-day mortality in COVID-19 patients on mechanical ventilation (13). However, there are still many studies that have shown no beneficial effects of GCs use in patients with severe pulmonary infection or ARDS (14). For example, GCs use within 3 days of admission to the ICU has been shown to be associated with increased 90-day mortality in patients with COVID-19 (15). Applying the joint model (JM) to longitudinal and time to event data has become a valuable follow-up data analysis tool (16) that combines a linear mixed model with a Cox proportional hazard model based on trajectory function for longitudinal data and time-event data. Compared to the individual model, the JM provides a more effective way to predict how event status (survival) was impacted by treatment and estimate how longitudinal data, usually be as biomarkers, was impacted by treatment. In addition, the JM can induce the correlation between the biomarker and the event status. Compared to the individual model, JM can reduce the overall prediction bias and provides more accurate results. Therefore, this study intends to extract ARDS patients from the public eICU Collaborative Research Database (eICU), and use JM to investigate the effects of GCs on the level of PaO2/FiO2 and survival of patients.

## Materials and Methods

### Data Source

The data analyzed in this study were extracted from the eICU Collaborative Research Database, which is a public multicenter intensive care unit (ICU) database containing data on over 200,000 patients (17, 18). As a multi-center database, eICU contains electronic medical records among 208 hospitals from 2014–2015. All information about patient's identity is hidden, so the patient's informed consent is not required. The author had participated in a series of courses provided by NIH and obtained authorization to access the eICU database after passing the required assessment (certificate number 38601114). All data were extracted using SQL (Structured Query Language) before further analysis. The variables were longitudinal measurements of PaO2/FiO2, age, sex, and race, BMI, first care unit, ventilator use, vasopressor use, continuous renal replacement therapy (CRRT), SOFA score, and APACHE IV score.

### Study Population

**Figure 1:**
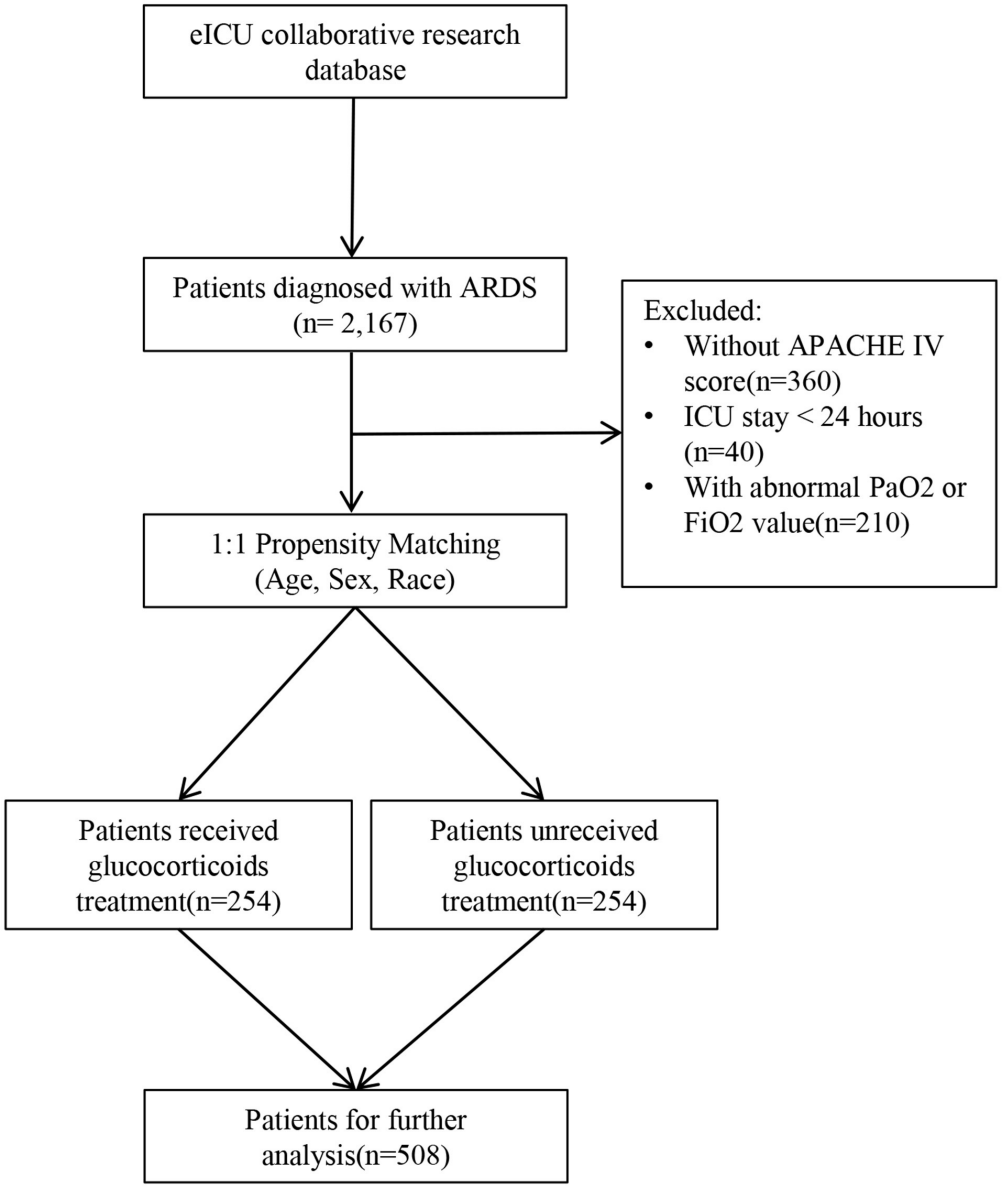
Inclusion and exclusion flowchart of the study.

This study initially included 2,167 patients diagnosed with ARDS. After excluded patients without an APACHE IV score, patients with ICU stay shorter than 24 h and patients with abnormal PaO2 or FiO2 values, 1,557 patients remained, among which 254 received GCs. After 1:1 propensity score matching (PSM) for GCs treatment status based on demographic data including age, sex, and race, 508 patients were eventually selected for the study (Figure 1). The primary study outcome was ICU mortality, the secondary outcome was the effect of GCs on PaO2/FiO2.

## Statistical Analysis

### Longitudinal Data Analysis in JM

The longitudinal data were analyzed by linear mixed effect model (Formula.1) in which Y_ij_ represents the longitudinal outcome value of ith patient at the jth time of observation. X_ij_ represents the trajectory of X of ith patient at the jth time of observation and R_ij_ is the random effect of ith patient at the jth time and K's represented other functions of co-variants of Y while ξ represents the coefficient (16). Every PaO2/FiO2 measurement was observed for each patient before death or ICU discharge. Linear mixed-effects models were modeled by the trajectory function and random effects to analyze the longitudinal data. The dependent variable was PaO2/FiO2 levels, and the independent variables were PaO2/FiO2 at admission, GCs treatment and its two-way interaction with observational time to accurately reflect the relationship between GCs treatment and trend of PaO2/FiO2 level for each patient (16). Adjustments of random effects were set as the observational time in order to minimize the random noises.

(1) $$\begin{array}{l} Y_{\text{ij}}=X_{\text{ij}}+\xi K_{\text{i}}+R_{\text{ij}} \end{array}$$

### Trajectory Function in JM

Sub-models in JM are combined by trajectory function (Formula.2). The X_ij_ represents the trajectory of longitudinal biomarker and the Z_i_ represents the treatment (GCs) for ith patients. The trajectory function represents the linear relationship between observation times for patients t_ij_ + treatment and the trajectory of biomarker (PaO2/FiO2). C_0i_ and C_li_ are considered to be random.

(2) $$\begin{array}{l} X_{\text{ij}}=C_{0\text{i}+}C_{\text{li}}\times t_{\text{ij}}+\gamma Z_{\text{i}} \end{array}$$

### Time-to-Event Data Analyses in JM

The time-to-event data were analyzed by the Cox proportional-hazards model (Formula.3). The H(t) represents the hazard function of ith patient at t, H_0_(t) represents the baseline hazard at t. β and α represents the coefficient of trajectory for ith patient and coefficient of treatment indicator at for ith patients and K's represents other co-variables and τ′*s* represents the coefficents.Compared to traditional Cox regression (Formula.4), the JM model not only reflects the relationship between treatment and both event and longitudinal biomarker, but also induces coefficient between biomarker and events. To determine the association between GCs treatment and ICU mortality. Cox proportional risk regression analysis was adjusted for imbalance variables between the two groups.

(3) $$\begin{array}{l} H\left(t\right)=H_{0}\left(t\right)exp\left(\beta X_{\text{ij}}+\alpha Z_{\text{i}+}\tau K_{\text{i}}\right) \end{array}$$

(4) $$\begin{array}{l} H\left(t\right)=H_{0}\left(t\right)exp\left(\alpha Z_{\text{i}+}\tau K_{\text{i}}\right) \end{array}$$

### Marginal Structural Cox Model

Whether or not patients with ARDS received GCs during ICU hospitalization was considered a time-dependent variable in MSCM. Potential baseline confounding factors such as age, sex, race, BMI, ICU unit type, ventilator use, vasopressor use, CRRT, SOFA, and Apache IV were obtained within 24 h of admission to the ICU. PaO2/FiO2 throughout ICU hospitalization was included in the model as a time-varying confounding factor. The parameters of the MSCM can be estimated using inverse probability of treatment weighing (IPTW). The gradient boosted model (GBM) is a machine learning algorithm that involves an iterative process using multiple regression trees to provide more accurate estimates of response variables (19). IPTW based on this algorithm has been proved to be effective and robust in the study of two treatments. Therefore, in this paper, we use this algorithm to weight each patient and generate two virtual populations. The standardized mean differences (SMDs) <0.2 between the two groups was considered to have no significant difference in baseline characteristics (20). Then Cox regression was conducted again to further prove the validity of the results. Continuous variables were described as median and interquartile values based on their normality and *P*-values were calculated by Kruskal–Wallis test. Categorical variables were described as number and percentage values, with *P*-values calculated using chi-square tests. All statistical analyses were conducted using R software, the JM was constructed using the “JM” package, and the IPTW was constructed using the “twang” package. A two-side *p*-value < 0.05 was considered statistically significant.

## Results

### Patient Characteristics

**Figure 2:**
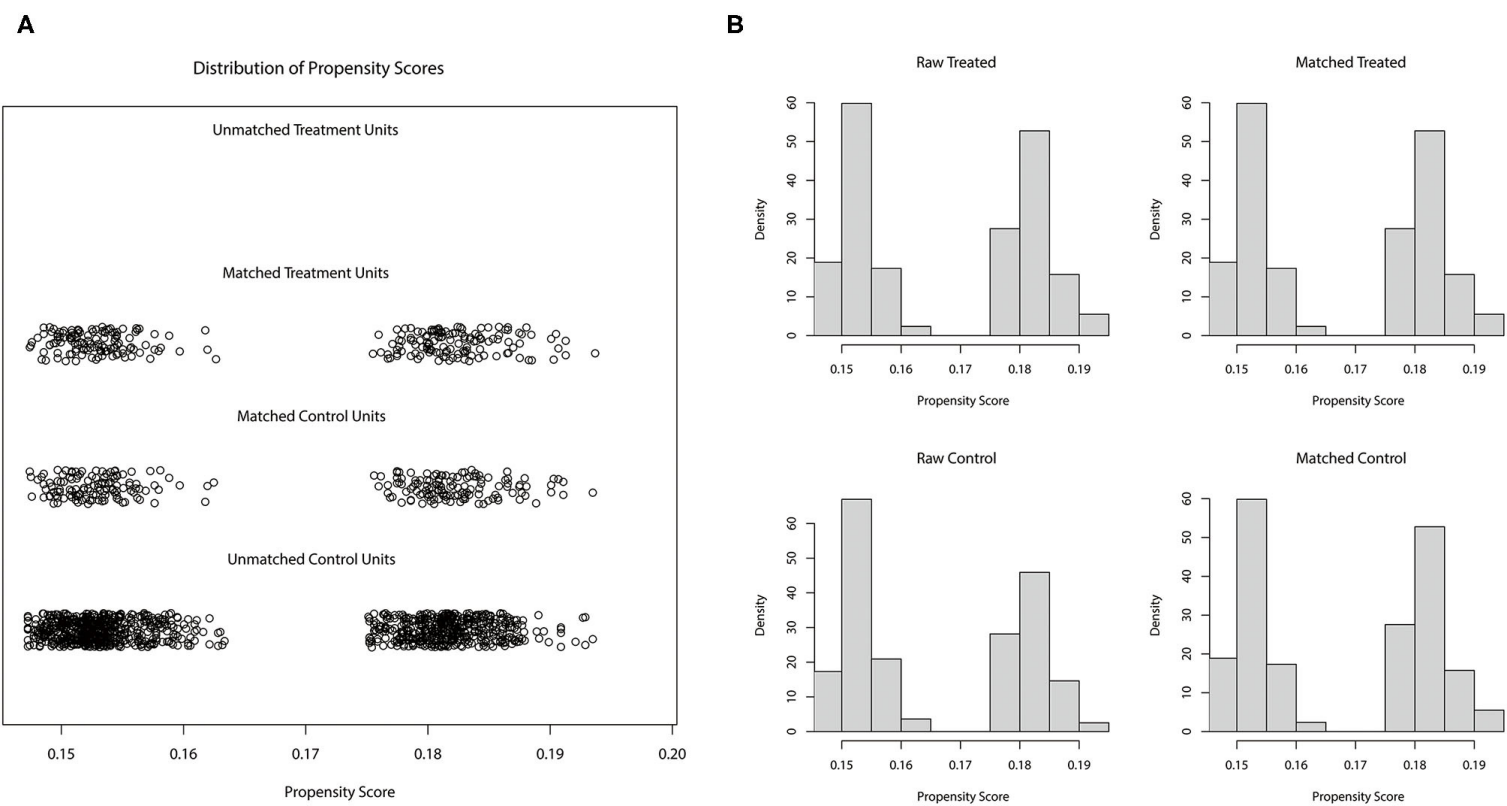
Distribution of 1:1 propensity matching score in cluster **(A)** and histogram **(B)** match was based on demographic information included age, sex, and race.

Among the 508 ARDS patients after PSM, 254 patients received GCs treatment (Figure 2). Table 1 lists the baseline characteristics of the between GCs groups. The result showed that after the match for demographic information, patients between GCs groups were different from first admitted PaO2/FiO2 and ventilator use.

**Table 1:** Baseline characteristics between GCs unreceived/received group.

	**GCs unreceived**	**GCs received**	***p***
*N*	254	254	
Age (year)	60.00 (49.00, 69.00)	59.00 (49.00, 69.00)	0.655
Sex (%)			1.000
Male	125(49.2)	125(49.2)	
Female	129 (50.8)	129 (50.8)	
Race (%)			0.080
White	189 (74.4)	178 (70.1)	
Black	21(8.3)	37 (14.6)	
Others	44 (17.3)	39 (15.4)	
BMI	28.76 (24.39, 36.81)	29.10 (24.52, 35.58)	0.770
First care unit (%)			0.235
CICU^a^	40 (15.7)	44 (17.3)	
SICU^b^	174 (68.5)	154 (60.6)	
MICU	33 (13.0)	48 (18.9)	
NICU^c^	7(2.8)	8(3.1)	
Ventilator (%)			
no	101 (39.8)	42 (16.5)	<0.001
yes	153 (60.2)	212 (83.5)	
Vasopressor (%)			
no	140 (55.1)	124 (48.8)	0.183
yes	114 (44.9)	130 (51.2)	
CRRT (%)			0.247
no	214 (84.3)	203 (79.9)	
yes	40 (15.7)	51 (20.1)	
First PaO2/FiO2	134.77(83.00,219.76)	113.06(75.00,170.00)	0.004
Apache IV score	75.50 (60.25, 96.75)	78.50 (58.00, 99.75)	0.680
SOFA score	8.00(6.00,11.00)	7.00(6.00,10.00)	0.155

a *include Cardiac ICU, CCU-CTICU, CTICU, CSICU;*

b *include Med-sug ICU, SICU;*

c *represent Neuro ICU*.

### Joint Model

**Figure 3:**
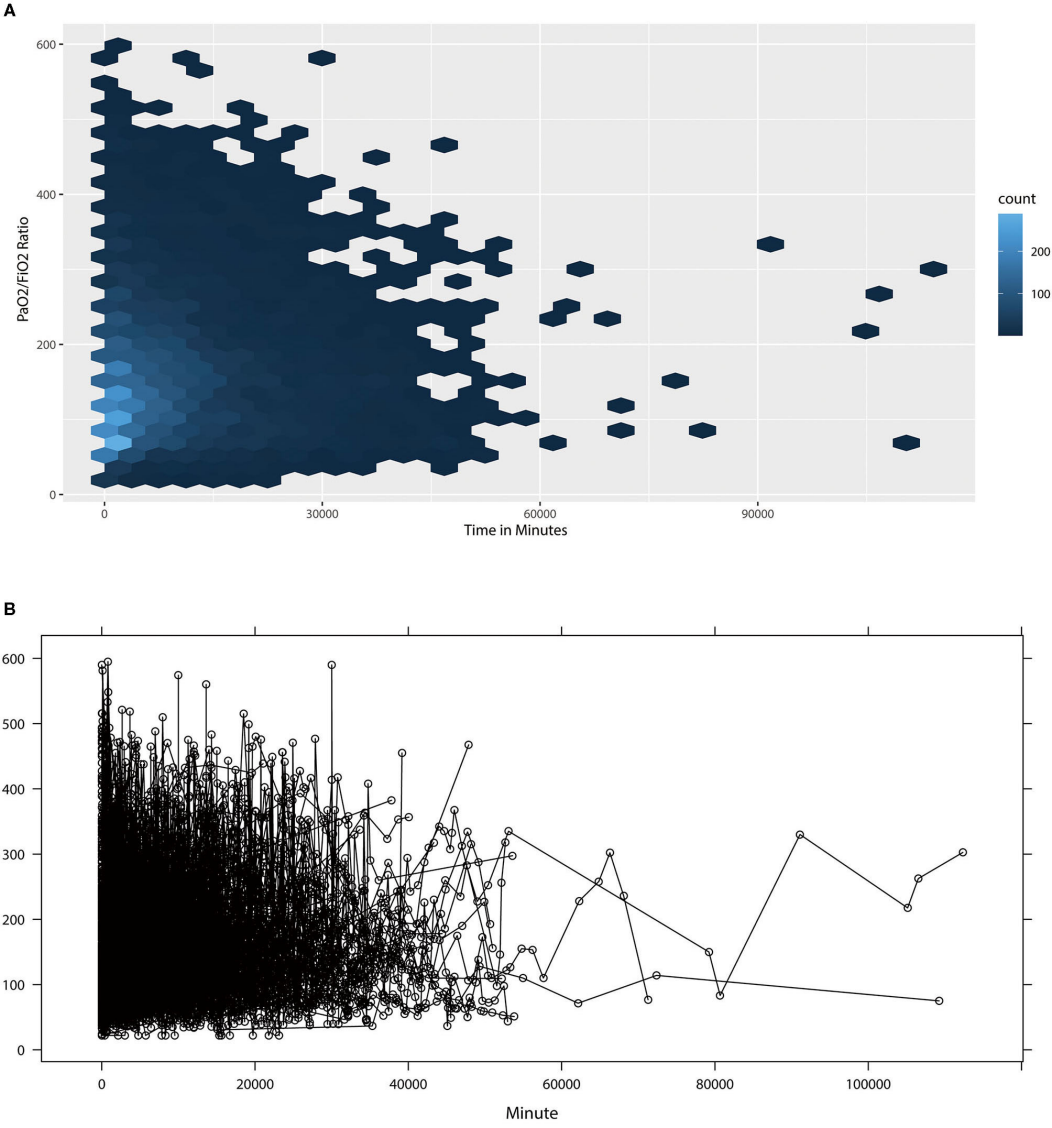
The distribution of PaO2/FiO2 observations **(A)** and the trajectory **(B)** of PaO2/FiO2 Observations by Patients in Time Line. **(A)** showed the density of PaO2/FiO2 ratio observations in time lines. **(B)** showed then trajectory of PaO2/FiO2 for 508 patients, each line represented the one patient's trajectory.

**Figure 4:**
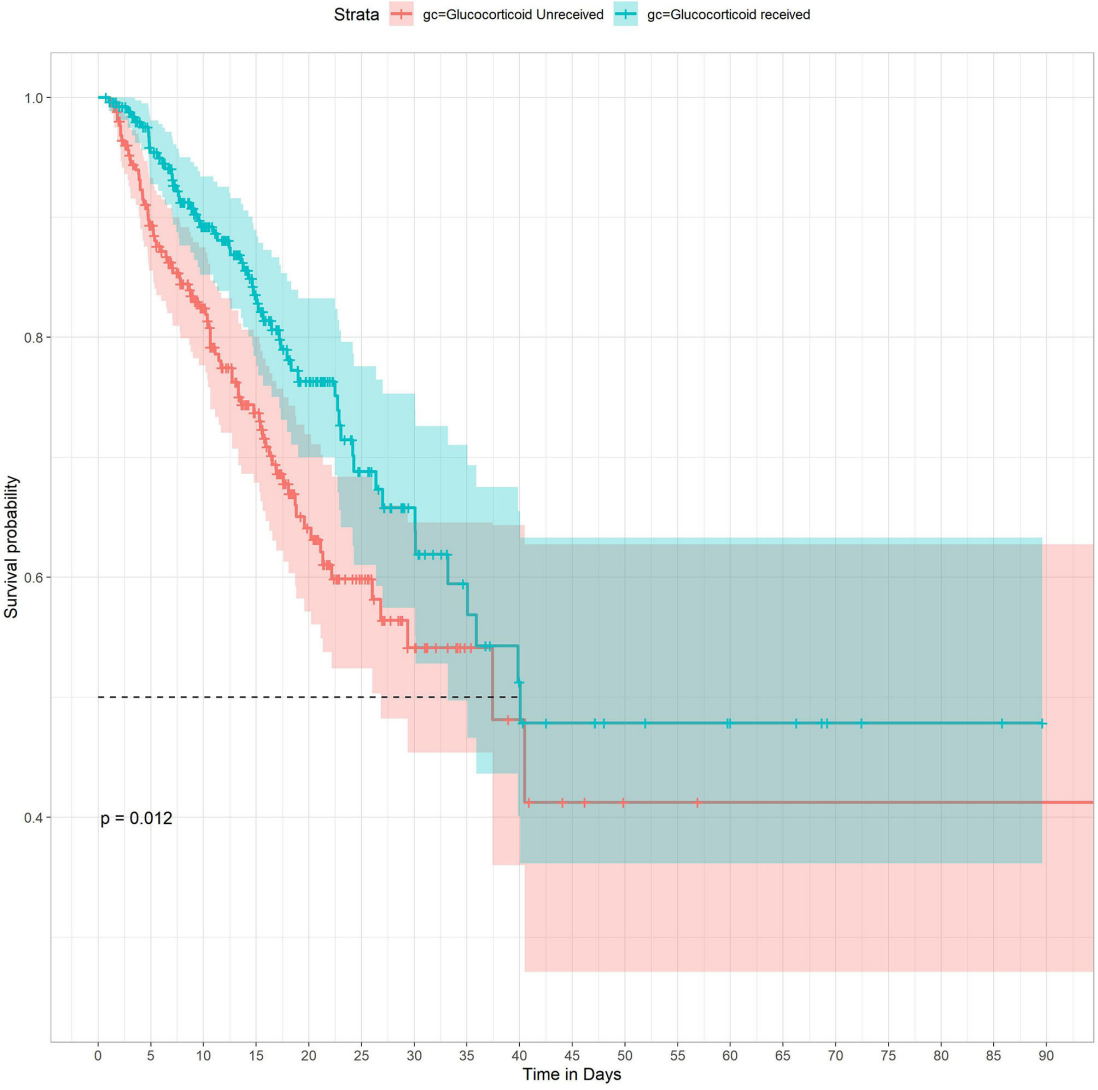
Kaplan-Meier survival curves between groups. *P*-value calculated by Log-rank test = 0.012 showed GCs received group had higher survival probability.

The distribution of longitudinal observation of 7,789 PaO2/FiO2 measurements can be shown using trajectory function and plotted using interaction figures. Figure 3 shows the density of PaO2/FiO2 values and the trajectory of each patient in different time periods. This plot indicates that most observations were concentrated in the first 5 days after patient admission. The result of multivariable Cox regression demonstrated that GCs treatment was associated with ICU mortality in ARDS patients [HR (95% CI) =0.642 (0.453, 0.912)], demonstrating that receiving GCs treatment is a protective factor in ARDS patients. The Survival curve also showed that GCs received group had lower risk of ICU mortality (*P* = 0.012) (Figure 4). Linear mixed-effects models demonstrated that GCs treatment was not correlated to PaO2/FiO2 trajectory after controlled by potential confounders. The result revealed that GCs had no effect on trend of PaO2/FiO2 level. The JM combined the linear mixed-effects model and the Cox regression model to a less biased and fixed result for both models. The results indicated that after the adjustment, GCs treatment was still a protective factor for ARDS patients (HR = 0.602), indicating that patients who received GCs treatments had 39.8% lower risk of mortality. In the JM, GCs was still not related to PaO2/FiO2. The JM also demonstrated an association between PaO2/FiO2 and the event status (HR = 0.991), indicating that one unit increase in PaO2/FiO2 will lower 0.9% of the ICU mortality risk of ARDS patients (Table 2).

**Table 2:** Result of effects between two sub-models, joint model and marginal structural cox model.

	**COX**		**LME**		**JM**		**MSCM**	
	**HR (95%CI)**	***P***	**HR (95%CI)**	***P***	**HR (95%CI)**	***P***	**HR(95%CI)**	***P***
Treatment effect on survive	0.642 (0.453, 0.912)	0.013			0.602(0.416–0.870)	0.007	0.597 (0.552,0.646)	<0.001
Treatment effect on PaO2/FiO2			0.001(0.000–11.491)	0.150	0.000(0.000–1.359)	0.060		
PaO2/FiO2 effect on survival					0.991(0.987–0.995)	<0.001		

*Cox, cox proportional hazards model; LME, linear mixed effect model; JM, joint model; MSCM: marginal structural cox model.*

*Coef, coefficient; SE, standard error. The hazard ratios (HRs) and 95% confidence intervals (error bars) were calculated from Coef and SE.*

*Cox sub-model included: First admitted PaO2/FiO2 level and ventilator use.*

LME sub-model included: GCs^*^time, first admitted PaO2/FiO2 level, ventilator use.

*MSCM included: Age, Sex, Race, BMI, First care unit, Ventilator use, Vasopressor use, CRRT, SOFA score, Apache IV score, overall PaO2/FiO2*.

### Marginal Structural Cox Model

In our study, the MSCM included patients' overall PaO2/FiO2, GCs treatment during ICU hospitalization, and baseline characteristics within 24 h after admission. SMDs after IPTW were <0.2 in both groups of virtual population. Baseline characteristics were shown in Supplementary Table 1. MSCM results showed that GCs use was associated with a significant improvement in ICU mortality in the ARDS population [HR (95%CI) = 0.597 (0.552, 0.646); *P* < 0.001]. See Table 2.

## Discussion

In the present study, although the GCs was not significantly associated with the improvement of PaO2/FiO2, under the Cox sub-model, the JM, and the MSCM results showed that receiving GCs may reduce the ICU mortality of patients with ARDS. And the longitudinal sub-model showed that increased PaO2/FiO2 also had beneficial effects on the survival of ARDS patients. There is extensive evidence that the use of GCs can reduce systemic inflammation and accelerate the regression of ARDS, and it is also involved in adaptive lung repair and the improvement of extrapulmonary physiology (21, 22). The mechanism is that GCs can stimulate and promote the apoptosis of helper T cells, inhibit the production of pro-inflammatory cytokines, reduce the snowball effect of inflammatory response from the source (23), inhibit adhesion expression molecules, prevent them from rolling and adhesion in the inflammatory site, and weaken the chemotaxis of neutrophils (24). CCs can also induce membrane coupling protein expression and promote the separation and apoptosis of neutrophils, therefore inhibiting the inflammatory response and induce macrophage gene expression (25), increase macrophage phagocytic activity, improve natural immune function, and inhibit the excessive proliferation of capillaries and fibroblasts and the onset of pulmonary fibrosis (26). There are many studies supporting the effectiveness of GCs in the treatment of ARDS, for example, a multi-center study showed that early administration of GCs can reduce the duration of mechanical ventilation and overall mortality in patients with confirmed moderate to severe ARDS (27). A meta-analysis of 1703 COVID-19 patients showed systemic corticosteroid administration was associated with lower 28-day all-cause mortality (28). So GCs seem to be a treatment for acute respiratory distress syndrome. The oxygenation index is a vital sign for the diagnosis of ARDS. The Berlin definition is classified by hypoxia severity, suggesting that more-severe hypoxia increases the probability of mortality and the survival time with mechanical ventilation (29). JM result indicated similar trends, with the risk of death decreasing by 0.9% for each unit increase in PaO2/FiO2. In our study, the use of GCs had no effect on PaO2/FiO2 trends but the reduced 39.8% of mortality rate. Plenty of factors affect the oxygenation index, the most common factors include the concentration of inhaled oxygen and PEEP (30). In patients with ARDS, the primary goal of GCs therapy is to improve pulmonary and systemic inflammatory conditions, and the improvement of PaO2/FiO2 may also require ventilator and other comprehensive treatments. Therefore, combining the GCs treatment and rational management of PaO2/FiO2 could significantly increase the survival of ARDS patients.

## Strengths and Limitations of the Study

As a multi-center study, the results of this study are more representative and reliable. The matching results of PSM show that confounding factors are well-adjusted. Compared with independent Cox regression, JM has less bias and more accurate correlation coefficients, so the results of this article have higher reliability. In addition, we use the MSCM to further verify the results. Of course, this study only also had limitations. This research only analyzed whether patients received GCs treatment but did not specifically analyze the type, dose, and duration of GCs, which needs further analysis in the future.

## Conclusions

The rational use of GCs therapy can reduce the ICU mortality of ARDS. Although GCs cannot improve PaO2/FiO2, combining GCs treatment and increasing PaO2/FiO2 can better improve patient survival.

## Data Availability Statement

The datasets presented in this study can be found in online repositories. The data were available on the eICU Collaborative Research Database at https://eicu-crd.mit.edu/.

## Ethics Statement

The study was an analysis of a third-party anonymized publicly available database with pre-existing institutional review board (IRB) approval.

## Author Contributions

LZ created the study protocol, performed the statistical analyses, and wrote the first manuscript draft. ZW conceived the study and critically revised the manuscript. FX assisted with the study design and performed data collection. YR assisted with data collection and manuscript editing. HW assisted the analysis and explain of statistical methods. DH confirmed the data and assisted with the statistical analyses. JL assisted with manuscript revision and data confirmation. HY contributed to data interpretation and manuscript revision. All authors read and approved the final manuscript.

## Conflict of Interest

The authors declare that the research was conducted in the absence of any commercial or financial relationships that could be construed as a potential conflict of interest.

## Publisher's Note

All claims expressed in this article are solely those of the authors and do not necessarily represent those of their affiliated organizations, or those of the publisher, the editors and the reviewers. Any product that may be evaluated in this article, or claim that may be made by its manufacturer, is not guaranteed or endorsed by the publisher.
