# Supplementary material for: The Role of Glucocorticoids in the Treatment of ARDS: A Multicenter Retrospective Study Based on the eICU Collaborative Research Database
Source: Front Med (Lausanne). 2021 Jul 26;8:678260. doi: 10.3389/fmed.2021.678260 (PMC8350484; doi:10.3389/fmed.2021.678260)
Supplement: Supplementary file 1 [file Table_1.DOCX]

Table S1 Baseline characteristics of the IPTW population.

|  | GCs Unreceived | GCs Received | SMD |
| --- | --- | --- | --- |
| N | 254 | 254 |  |
| Age (year) | 59.00 (46.00, 68.00) | 59.00 (49.00, 66.00) | 0.012 |
| Sex (%) |  |  | 0.035 |
| Male | 3417.3 ( 49.8) | 3640.1 ( 51.6) |  |
| Female | 3441.2 ( 50.2) | 3415.1 ( 48.4) |  |
| Race (%) |  |  | 0.146 |
| White | 5215.0 ( 76.0) | 4998.0 ( 70.8) |  |
| Black | 710.8 ( 10.4) | 1061.8 ( 15.0) |  |
| Others | 932.6 ( 13.6) | 995.4 ( 14.1) |  |
| BMI | 30.11 (25.28, 36.45) | 30.39 (25.18, 36.92) | 0.015 |
| First care unit (%) |  |  | 0.124 |
| CICU^a^ | 1111.8 ( 16.2) | 1393.3 ( 19.7) |  |
| SICU^b^ | 4576.5 ( 66.7) | 4364.3 ( 61.9) |  |
| MICU | 866.8 ( 12.6) | 1040.5 ( 14.7) |  |
| NICU^c^ | 303.5 ( 4.4) | 257.1 ( 3.6) |  |
| Ventilator (%) |  |  | 0.128 |
| no | 1548.6 ( 22.6) | 1233.1 ( 17.5) |  |
| yes | 5309.9 ( 77.4) | 5822.0 ( 82.5) |  |
| Vasopressor (%) |  |  | 0.049 |
| no | 3110.9 ( 45.4) | 3027.4 ( 42.9) |  |
| yes | 3747.6 ( 54.6) | 4027.7 ( 57.1) |  |
| CRRT (%) |  |  | 0.010 |
| no | 4825.1 ( 70.4) | 4930.6 ( 69.9) |  |
| yes | 2033.4 ( 29.6) | 2124.6 ( 30.1) |  |
| overall PaO2/FiO2 | 148.00 (99.20, 208.69) | 138.33 (95.56, 196.00) | 0.087 |
| Apache IV score | 79.00 (64.00, 103.00) | 83.00 (61.00, 108.00) | 0.048 |
| SOFA Score | 8.00 (6.00, 11.00) | 9.00 (6.00, 11.00) | 0.009 |

Note: a include Cardiac ICU, CCU-CTICU, CTICU, CSICU; b include Med-sug ICU, SICU; c represent Neuro ICU.
